# Pharmacokinetic-pharmacodynamic modelling of the cardiovascular effects of drugs – method development and application to magnesium in sheep

**DOI:** 10.1186/1471-2210-5-5

**Published:** 2005-03-10

**Authors:** Richard N Upton, Guy L Ludbrook

**Affiliations:** 1 Anaesthesia and Intensive Care, Royal Adelaide Hospital, North Terrace, Adelaide, SA 5000, Australia; 2 Anaesthesia and Intensive Care, University of Adelaide, North Terrace, Adelaide, SA 5005, Australia

## Abstract

**Background:**

There have been few reports of pharmacokinetic models that have been linked to models of the cardiovascular system. Such models could predict the cardiovascular effects of a drug under a variety of circumstances. Limiting factors may be the lack of a suitably simple cardiovascular model, the difficulty in managing extensive cardiovascular data sets, and the lack of physiologically based pharmacokinetic models that can account for blood flow changes that may be caused by a drug. An approach for addressing these limitations is proposed, and illustrated using data on the cardiovascular effects of magnesium given intravenously to sheep.

The cardiovascular model was based on compartments for venous and arterial blood. Blood flowed from arterial to venous compartments via a passive flow through a systemic vascular resistance. Blood flowed from venous to arterial via a pump (the heart-lung system), the pumping rate was governed by the venous pressure (Frank-Starling mechanism). Heart rate was controlled via the difference between arterial blood pressure and a set point (Baroreceptor control). Constraints were made to pressure-volume relationships, pressure-stroke volume relationships, and physical limits were imposed to produce plausible cardiac function curves and baseline cardiovascular variables. "Cardiovascular radar plots" were developed for concisely displaying the cardiovascular status. A recirculatory kinetic model of magnesium was developed that could account for the large changes in cardiac output caused by this drug. Arterial concentrations predicted by the kinetic model were linked to the systemic vascular resistance and venous compliance terms of the cardiovascular model. The kinetic-dynamic model based on a training data set (30 mmol over 2 min) was used to predict the results for a separate validation data set (30 mmol over 5 min).

**Results:**

The kinetic-dynamic model was able to describe the training data set. A recirculatory kinetic model was a good description of the acute kinetics of magnesium in sheep. The volume of distribution of magnesium in the lungs was 0.89 L, and in the body was 4.02 L. A permeability term (0.59 L min^-1^) described the distribution of magnesium into a deeper (probably intracellular) compartment. The final kinetic-dynamic model was able to predict the validation data set. The mean prediction error for the arterial magnesium concentrations, cardiac output and mean arterial blood pressure for the validation data set were 0.02, 3.0 and 6.1%, respectively.

**Conclusion:**

The combination of a recirculatory model and a simple two-compartment cardiovascular model was able to describe and predict the kinetics and cardiovascular effects of magnesium in sheep.

## Background

The effective use of some drugs can be limited by their adverse effects on the cardiovascular system, particularly when they are used intravenously in relatively high doses. There have been many studies documenting the cardio-vascular effects of drugs. Similarly, many mathematical models of the cardiovascular system, of varying complexity, have been presented in the literature [1,2]. In pioneering work, models of the cardiovascular system have been linked to pharmacokinetic models of volatile anaesthetic disposition [3-5]. These kinetic-dynamic models have since been developed into mannequin based anaesthesia simulators, which now have a pivotal role in the training of anaesthetists. This approach has been facilitated by the fact that models of volatile anaesthetic disposition have traditionally been physiologically based (e.g. using representations of tissue:blood partition coefficients and blood flows for individual organs or groups of organs). It is therefore possible to equate blood flow in the cardiovascular model to blood flow in the pharmacokinetic model. Nevertheless, a limiting factor in the implementation of this approach is the availability of experimental data on concentration-effect relationships [5].

In contrast, for traditional ("non-volatile") drugs, there have been very few instances in which kinetic models of a drug have been linked to cardiovascular pharmacodynamic models. The work of Francheteau et al. is an important exception [6], but even this early work was restricted to analysis of only one aspect of the cardiovascular system (i.e. accounting for heart rate mediated control of blood pressure but not Frank-Starling control of cardiac output). However, it is clear this approach has the potential to provide a more rational basis for designing dose regimens of cardio-active drugs, and could provide insight into strategies for controlling their cardio-vascular effects. It maybe possible to predict *a priori *the cardiovascular consequences of, for example, a change in dose regimen of a drug.

There are a number of difficulties in implementing this approach for traditional drugs. One problem is that most drugs do not cause changes in one single cardiovascular variable (such as blood pressure) that can be described in the usual manner using a simple semi-empirical dynamic model (e.g. an E_max _model). Rather, a number of cardiovascular variables may be altered simultaneously in a manner that is complex and interrelated. Thus, any dynamic model used must account for these intrinsic relationships between cardiovascular variables. Another problem is that changes in the cardiovascular system (in particular blood flow distribution) invariably alter the kinetics of the drug under study. Therefore, the kinetic model of the drug must be able to account for the effects of blood flow changes on the disposition of the drug. This requires the kinetic model to have a physiological basis, and importantly excludes the widely used mamillary compartmental pharmacokinetic model.

The general aims of this study were threefold. First, to develop a simple dynamic model of the cardiovascular system that was of sufficient complexity to account for the major mechanisms by which drugs can alter cardiovascular variables. Second, to examine whether recirculatory kinetic models [7] have sufficient physiological basis to account for drug related blood flow changes. Third, to examine approaches for identifying the important concentrations (and their sites in the body) that can be used to link the kinetic and dynamic models.

The specific aim was to use previously published data collected using a chronically instrumented sheep preparation [8,9] to develop a kinetic-dynamic model for the cardiovascular effects of magnesium. Magnesium is given intravenously to treat a number of diseases, including pre-eclampsia. It relaxes smooth muscles in blood vessels thereby lowering systemic vascular resistance, with a consequent decrease of mean arterial blood pressure and increase in cardiac output. It provides a useful drug for initial analysis as its kinetics and dynamics are relatively simple and well understood.

The overall hypothesis of this work is that it is possible to construct a faithful model of the cardiovascular effects of drugs such as magnesium. While doing so requires more assumptions and estimates of parameter values than normally associated with semi-empirical pharmacokinetic-pharmacodynamic modelling, a physiological approach greatly increases the utility of the resulting models. It is proposed that the general methods presented here could be applied to the development of similar models for other drugs with acute cardiovascular effects.

## Methods

### General rationale

With respect to devising a pharmacodynamic model of the cardiovascular system, the important steps are:

1. Identifying which cardiovascular variables (e.g. heart rate, blood pressure) are important. This depends on the drug and the intended use of the model, but it is proposed that there is a minimum set of variables that is needed for a basic description of cardiovascular status.

2. Devising a way of conveniently presenting the output of the dynamic model for a range of cardiovascular variables for comparison with data.

3. Identifying a cardiovascular model of the appropriate complexity. Ideally the model must be of the minimum complexity that includes the cardiovascular variables identified above, and the major sites of action of the drugs.

4. Identifying which parameters of the cardiovascular model can be estimated by curve-fitting, and which require prior estimates or measurements of physiological values. Most cardiovascular models are stiff numerical systems with many parameters, and only a small number can be estimated by curve-fitting the data in the traditional way.

With respect to the pharmacokinetic model, there remains one crucial step:

5. Constructing a kinetic model with a physiological basis that is sufficiently realistic to describe and predict the concentration of the drug in the key target organs controlling the cardiovascular system. On first principles, these could be expected to include:

a. the myocardial concentrations when the drug has a direct myocardial effect (e.g. causes myocardial depression);

b. the CNS concentrations when the drug affects the cardio-respiratory control centre of the brain;

c. the arterial blood concentration when the drug affects baro-receptors or smooth muscle in the walls of the arterial vascular system;

d. the venous blood concentration when the drug affects smooth muscle in the wall of the capacitance vessels of the venous vascular system.

It is known that these concentrations can follow different time-courses, particularly after bolus administration or a change of infusion rate [10,11]. However, it may not be necessary to know the time-course of these concentrations for every drug, depending on its mechanism of action.

### Data sets and software

The data used to construct the model were collected in the same laboratory using a conscious chronically instrumented sheep preparation and have been published previously [8,9]. This facilitated the model building process, as the effect of differences in species and measurement methods could be discounted.

Data set 1 [9] (for model development) was a detailed set of cardiovascular measurements made after the administration of 30 mmol of magnesium over 2 min to 5 sheep. Measurements included arterial and coronary sinus (effluent from the heart) magnesium concentration, cardiac output, mean arterial blood pressure, heart rate, an index of myocardial contractility (Maximum positive rate of change of left ventricular Pressure, dp/dt) and an index of filling pressure (Left ventricular end diastolic pressure) and myocardial blood flow. These were made until 25 min after the start of administration.

Data set 2 [8] (for model validation) was a less comprehensive set of cardiovascular measurements made after the administration of 30 mmol of magnesium over 5 min to 5 sheep (not the same sheep as Data set 1). Measurements included arterial magnesium concentration, cardiac output, and mean arterial blood pressure, and were made until 25 min after the start of administration. The blood pressure data for one animal in this data set was excluded, as it was idiosyncratically low.

The time-course of the data averaged across sheep were used for all modelling – the resultant model therefore represents the response of the average sheep. Inter- and intra-animal variability were not considered, although it is noted that the final model may provide insight into sources of kinetic and dynamic variability for later study.

The software used was the Scientist for Windows program (Version 2.01, Micromath, Salt Lake City, Utah, USA), predominantly for curve-fitting. The R language, Version 1.9.0, [12] was used for graphical data analysis, data handling and simulations. Coding the same model in the two different programs provided a useful check for errors.

For the least squares curve-fitting, the best fit was determined as that with the highest Model Selection Criteria (MSC) and without non-identifiable parameters. The MSC is essentially an inverse Akaike Information Criterion scaled to compensate for data sets of different magnitudes (Scientist for Windows manual, Micromath, Salt Lake City, Utah, USA), and is calculated as follows where w_i _is a weighting term, p is the number of parameters and n is the number of data points:



All data points were weighted equally. A parameter was arbitrarily defined as non-identifiable if the standard deviation of the parameter returned by the fitting program was greater than the parameter estimate (i.e. the coefficient of variation was greater than 100%). A model with non-identifiable parameters means that the data do not contain sufficient information to estimate the parameter with precision.

The symbols used throughout have been based on standards for the pharmacokinetic literature. Unfortunately, the use of C for both concentration (in pharmacokinetics) and compliance (in cardiovascular physiology) creates of conflict for this paper. To avoid confusion, CPL will be used for compliance here.

### Pharmacodynamic model of the cardiovascular system

#### Identification of important cardiovascular variables

The choice of the cardiovascular variables used in the model is clearly dependent on the drug under study and the intended purpose of the model. However, we propose that a minimum of 7 fundamental cardiovascular variables is sufficient for most pharmacological purposes. These variables are shown with their default unit of measurement in the model: Central venous pressure (CVP, mmHg), Myocardial contractility (CNT, L mmHg^-1^), Stroke volume (SV, L), Heart rate (HR, min^-1^), Cardiac output (CO, L min^-1^), Systemic vascular resistance (SVR, Resistance units, RU) and mean arterial blood pressure (MAP, mmHg).

This choice of variables requires several assumptions:

##### Assumption 1

All variables are time averaged in that beat to beat variation is ignored (e.g. mean arterial blood pressure is used rather than systolic and diastolic blood pressures).

##### Assumption 2

That the function of the left and right side of the heart is the same, and there are no abnormalities in the pulmonary vasculature so that the heart-lung system can be treated as one pump.

##### Assumption 3

Long time-scale events such as fluid shifts and renal mechanisms controlling blood pressure are ignored.

Furthermore, this choice of variables is dictated by several fundamental relationships between the variables. Firstly, that myocardial contractility is a proportionality constant between CVP pressure and stroke volume (the volume of blood pumped with each beat of the heart).

CVP * CNT ≈ SV    ...(2)

Mathematically, CNT must therefore have the units of volume / pressure. However, contractility is difficult to measure in vivo, and that there are a number of surrogate measures including dp/dt. These can also be used with appropriate scaling factors.

##### Assumption 4

That there are no factors affecting the relationship between myocardial fibre length (the true determinant of stroke volume) and central venous pressure (e.g. changes in myocardial compliance). CVP is therefore used as an easily measured index for myocardial fibre length – the assumption is that the two are related using a scaling factor. Left ventricular end diastolic pressure is also as an alternative index when data are presented as percent change from baseline.

The second relationship is that between stroke volume and heart rate to give the cardiac output (the volume of blood pumped by the heart per unit time):

SV * HR = CO    ...(3)

The last relationship is that between cardiac output and systemic vascular resistance to approximate the mean arterial blood pressure.

CO/SVR ≈ MAP    ...(4)

This is because MAP usually greatly exceeds the CVP:

CO/SVR = MAP-CVP    ...(5)

SVR therefore has the units of pressure over flow. In this paper, the resistance units (RU) are therefore mmHg L^-1 ^min.

### Presentation of relationships between cardiovascular variables

The effect of a drug on one or two variables can usually be summarised on a plot of the variable (drug effect) against time. However, it is more difficult to summarise the dynamic effect of a drug on the cardiovascular system for the following reasons: First, the large number of variables required in the summary, where the seven described above could be considered a minimum. Second, the fixed inter-relationships (e.g. Eqns. 2–4) between these variables that should be revealed by the summary (e.g. if SV increased by 25% and all else remains the same, then CO should also increase by 25% (Eqn. 3)). Third, usually an analysis requires comparing one cardiovascular state (e.g. pre-drug) with another (e.g. post-drug), or examining the time-course of drug effects.

**Figure 1:**
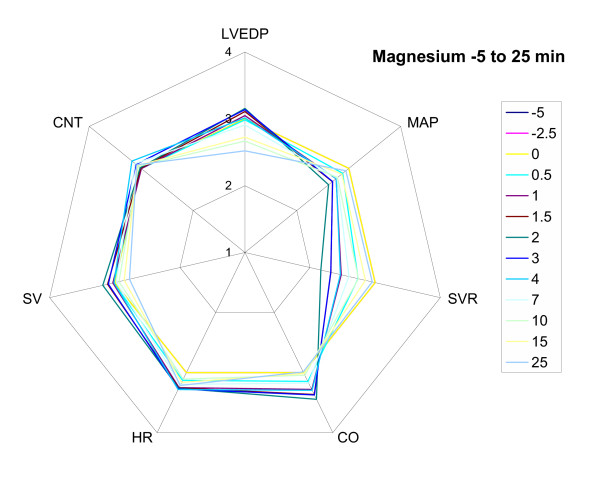
**An example of a cardiovascular radar plot. **A cardiovascular radar plot of the effect of magnesium (n = 5 sheep) on the cardiovascular system under baseline conditions and for a number of time-points until 25 min after the intravenous administration of magnesium. A scale for each of the 7 key cardiovascular variables radiates from the centre of the plot. LVEDP (left ventricular end diastolic pressure) is a surrogate for CVP; both should change proportionally. The data are transformed and the scale constructed so that 3 is the baseline (pre-drug) value. Thus, the blue line for the baseline data is a ring passing through 3 for each variable. Baseline conditions therefore have a characteristic equilateral 7 sided shape. The full scale is structured as follows: 1 one quarter baseline 2 half baseline 3 baseline 4 twice baseline 5 4 times baseline This scale has the property that for an equivalent increase or decrease in a cardiovascular variable compared to baseline, the line will move an equal distance in or out from the baseline value. It can be seen that following magnesium there was a drop in SVR with a baroreflex increase in HR to compensate for the drop in blood pressure. LVEDP also dropped, but with minimal change in contractility. Drugs that affect the cardiovascular system via different mechanisms produce plots with characteristic shapes, which can be recognised with experience. Note: The order of the variables on the radar plot has been chosen to account for key relationships between the variables, with CVP (as given by LVEDP) as the most fundamental variable at the top: In an anti-clockwise direction the following relationships or approximations hold: CVP * CNT ≈ SV SV * HR = CO CO/SVR ≈ MAP

It is proposed that a "cardiovascular radar plot" with a modified logarithmic, normalised scale is an efficient means of limiting these problems. An example radar plot is shown and described in Fig. 1. Radar plots are particularly useful for visually testing whether a model of the cardiovascular system behaves appropriately for all 7 key cardiovascular variables when challenged with a particular drug or physiological circumstance. It is particularly useful to see if the pattern of changes is internally consistent. For example, in Fig. 1 it is clear that magnesium dropped SVR, but the drop in MAP was not great as expected because there was a baroreceptor mediated increase in heart rate.

### Cardiovascular model – Structure and parameter estimation

There are many published models of the cardiovascular system of various levels of complexity and intended for various tasks [1]. However, in this paper, the cardiovascular model was constructed progressively from first principles, with adaptations and increases in complexity as dictated by the requirements of the modelling process and the data. This ensured the model was the minimum that was needed for the task at hand.

In vivo, the cardiovascular system has two major control systems; control of cardiac output via the Frank-Starling mechanism, and control of blood pressure via baroreceptor control of heart rate. These were added progressively to the model.

#### A simple Frank-Starling model

**Figure 2:**
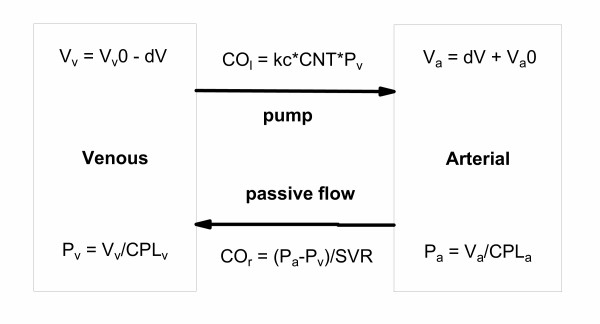
**A simple Frank-Starling model of the cardiovascular system. **A simple two compartment model of the circulation, with control of the cardiac output via the Frank-Starling mechanism. When the heart is not pumping, the pressures on the venous (P_v_) and arterial (P_a_) sides of the circulation are equal (the mean circulatory pressure (MCP = P_v _= P_a_) is approximately 7 mmHg). The unstressed volumes of the venous (V_v_0) and arterial sides (V_a_0) are governed by the relative compliance of the venous and arterial pools (CPL_v _and CPL_a_, respectively). If the pumping action of the heart is initiated, a fraction of the blood (dV) moves from the venous to the arterial side thereby increasing arterial pressure and decreasing venous pressure. The pressure gradient causes blood to flow from the arterial side to the venous side (at a rate given by the venous return, CO_R_). This depends on the pressure gradient (P_a_-P_v_) and the systemic vascular resistance (SVR).

A simple model of the Frank-Starling mechanism was developed (Fig. 2) assuming the blood is predominately in two pools – the arterial and venous vasculature. The two pools are connected by a pump (the heart) moving blood from the venous to arterial side for which the rate of pumping is proportional to the venous pressure. Blood flows from the arterial to venous side through a passive resistance (the SVR). The pressure in each pool is a function of the compliance in the pool. Compliance (CPL) governs the relationship between volume and pressure:

Pressure = Volume/Compliance    ...(6)

The solution to the simple Frank-Starling model can be found algebraically, but for consistency is shown in Additional File 1 as differential equations.

**Figure 3:**
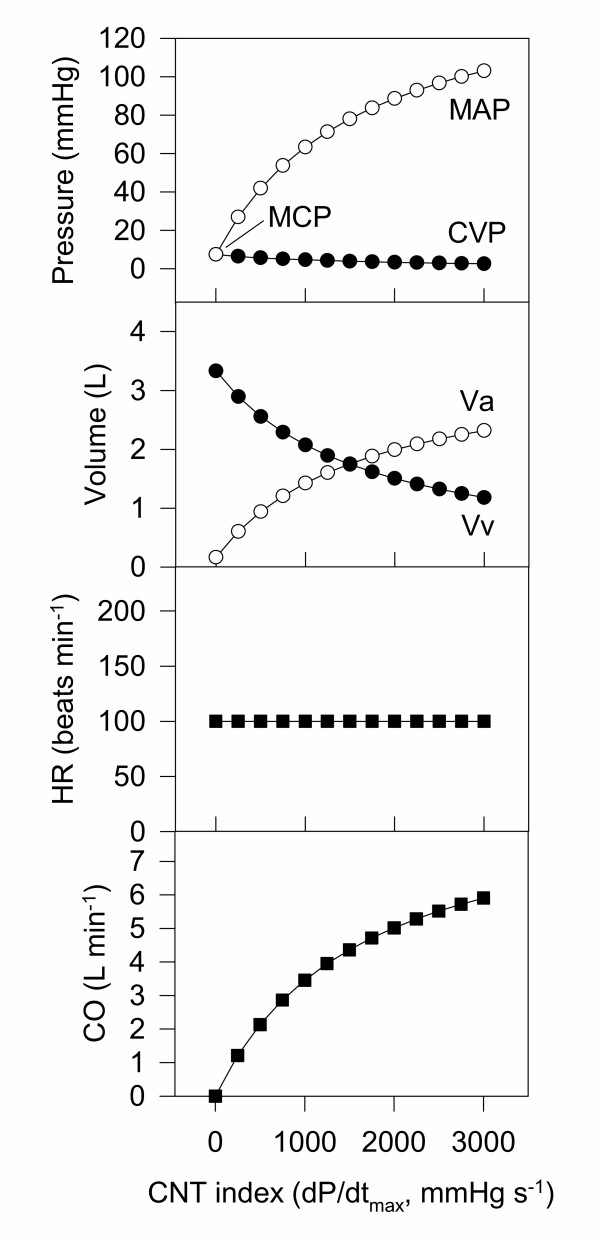
**Cardiac function curves for the Frank-Starling model. **A summary of the behaviour of the simple Frank-Starling model. The relationship between cardiac function (contractility) and the arterial and venous pressures matches well that reported in many textbooks. The venous compliance CPL_v _was 0.45, and the ratio of CPL_v_/CPL_a _was 15.

Central to the Frank-Starling model is the concept of cardiac function curves – usually given as the pressure in each pool as cardiac function (contractility) is increased from zero to a normal value. These curves are useful for finding appropriate initial estimates for blood volume, arterial and venous compliance, and systemic vascular resistance. To achieve the physiologically plausible cardiac function curve shown in Fig. 3, blood volume was set at 3.5 L [13]. Given that in a normal (50 kg) sheep the baseline cardiac output is approximately 6 L/min and mean arterial blood pressure is 100 mmHg (Table 1), baseline systemic vascular resistance is therefore 100/6 ≈ 17 RU. The remaining unknowns of this system (kc, CPL_a_, CPL_v_) were chosen to duplicate the following behaviour (Fig. 3) which is consistent with measurements in this species: When CNT is zero (i.e. the heart is not pumping) then dV is zero and the mean circulatory pressure (MCP) is approximately 7 mm Hg. When CNT is such that the cardiac output is approximately 6 L min^-1^, then MAP and CVP are approximately 100 and 2 mmHg, respectively (Table 1). In practice, it was found easier to express the arterial compliance (CPL_a_) as the ratio of arterial compliance to venous compliance (C_ratio_).

**Table 1:** Baseline (pre-drug) cardiovascular variables. A set of target values that was representative of the sheep studied in our laboratory was compiled from previous measurements and literature values as indicated. A set of parameter values for the final (Constrained-Frank-Starling-Baroreceptor) was derived (Table 2) that produced an internally consistent model that closely replicated these target values (also shown for comparison).

**Variable**	**Name**	**Target Value**	**Target value origin**	**Model derived Value**	**Units**
V_blood_	Blood volume	3.5	literature [13]	3.5	L
CVP	Central venous pressure	2.00	unpublished previous measurements and literature [20]	2.00	mmHg
CPL_v_	Venous compliance	0.45	inferred from V_blood _& CVP	0.46	L mmHg^-1^
MAP	Mean arterial pressure	100	previous measurements [21]	100.9	mmHg
SVR	Systemic vascular resistance	17.00	calculated from CO & MAP	17.0	RU
CO	Cardiac output	6	previous measurements [21]	5.8	L min^-1^
HR	Heart rate	100	previous measurements [21]	98.3	beats min^-1^
SV	Stroke volume	0.06	calculated from CO & HR	0.059	L
CNT	Contractility	3000	previous measurements [9, 21]	3000	mmHg sec^-1^
S1	Sympathetic tone – chronotropy	1	scaling factor only	1	dimensionless
S2	Sympathetic tone – Contractility	1	scaling factor only	1	dimensionless

#### Frank-Starling and Baroreceptor model

**Figure 4:**
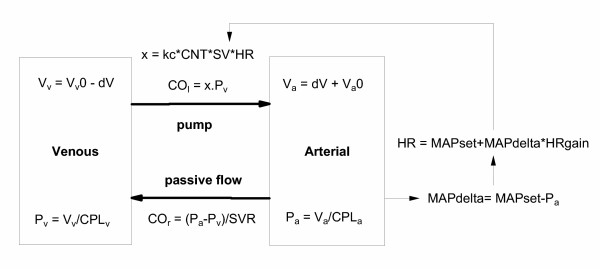
**A Frank-Starling-Baroreceptor model of the cardiovascular system. **The Frank-Starling model of the circulation from Fig. 2 combined with baroreceptor control of arterial blood pressure (P_a_) via changes in heart rate (HR). MAP_set _is the set point of the control system, and HR_gain _is the gain of the control system that operates on the difference between the actual and set arterial blood pressures (MAP_delta_; Eqn. 7). The right side cardiac output term is expanded to include the role of myocardial contractility (CNT), stroke volume (SV) and heart rate (HR). "kc" is a conversion factor to adjust for the index used to measure myocardial contractility (Eqn. 8). Strictly, myocardial contractility is the proportionality factor between Pv and stroke volume (SV = CO_R_/HR). However, it is often quantified using indirect indices, such as maximum positive change of ventricular pressure (dP/dt_max_). The value of kc will depend of what index of contractility is used (see Eqn. 8).

**Figure 5:**
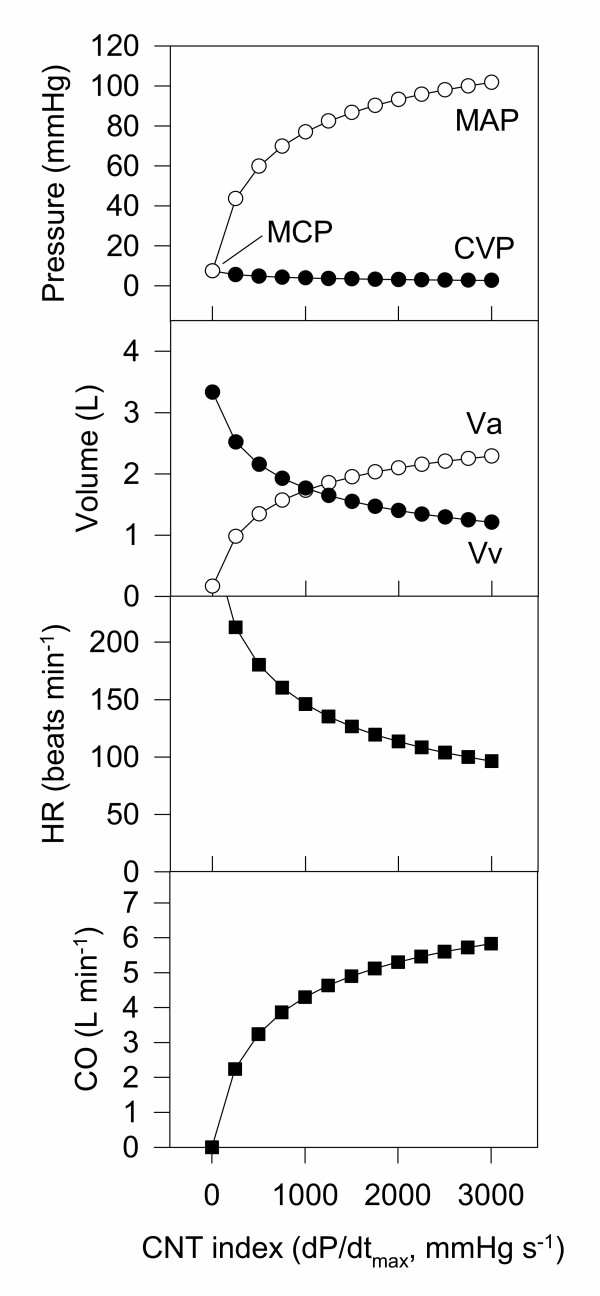
**Cardiac function curves for the Frank-Starling-Baroreceptor model. **A summary of the behaviour of the Frank-Starling-Baroreceptor model. The relationship between cardiac function (contractility) and the arterial and venous pressures matches well that reported in many textbooks. However, the vascular volumes show the majority of the blood in the arterial compartment, which is at odds with the fact that the majority of the blood under baseline conditions is in the venous vessels. Furthermore, no constraints have been placed on the model so that unrealistic values (e.g. large negative pressures) can be achieved in some circumstances.

The control of arterial blood pressure via baroreceptor control of heart rate was added to the simple Frank-Starling model, as shown in Fig. 4. The arterial pressure set point (MAPset) was used to calculate the difference between the actual and set pressure (MAPdelta). This pressure difference was used to change heart rate with a gain given by "HRgain". When HRgain is zero, the model reduces to the simple Frank-Starling model. As HRgain is increased, the more heart rate is adjusted to defend changes in arterial pressure. A value of 3 was initially used for HRgain. The resultant cardiac function curve for this model is shown in Fig. 5, and the equations for the model are shown in Additional File 2.

#### Constraining the model to increase physiological plausibility

**Figure 6:**
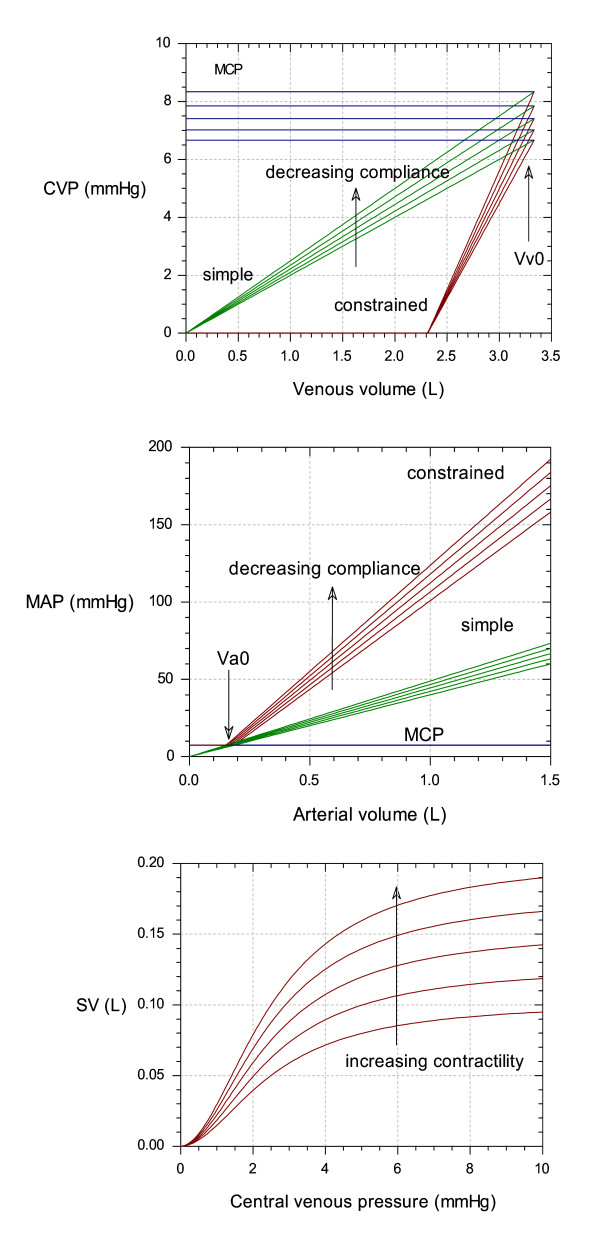
**Effect of introducing constraints on the model. **Top: Venous pressure – volume curves. Venous volume starts at V_v_0 when the heart is not pumping, at which point the venous pressure is the mean circulatory pressure (MCP). With increased pumping, the venous volume and venous pressure is reduced. In the simple (Frank-Starling-Baroreceptor) model, the CVP – volume relationship was linear, with an intercept of zero. In the constrained model, a lower intercept was used which was necessary to produce realistic venous volumes under baseline conditions. The multiple curves in the plot show the effect of changing venous compliance (CPL_v_). Middle: Arterial pressure – volume curves. Arterial volume starts at Va0 when the heart is not pumping, at which point the arterial pressure is the mean circulatory pressure (MCP). With increased pumping, the arterial volume and arterial pressure are increased. In the simple model, the MAP – volume relationship was linear, with an intercept of zero. In the constrained model, a lower intercept was the used, which was necessary to produce realistic arterial volumes under baseline conditions. The multiple curves in the plot show the effect of changing compliance (CPL_a_). Bottom: "Cardiac output curves" In this case cardiac output is given by stroke volume, which is plotted against central venous pressure (CVP). In the simple model, this relationship was linear. In the constrained model the relationship was given by a logistic equation which rose to a limit. The left-hand side of the curves are pseudo-linear, and the slope of the lines increase with increasing contractility. This behaviour mimics the "Cardiac output curves" found in many cardiovascular textbooks (e.g. Guyton [19]).

The final version of the model introduced a number of constraints to increase its physiological plausibility. These were: 1) Assuming that under baseline conditions that approximately 1/3 of the total blood pool is in the arterial system. 2) That the intercepts of the pressure-volume "curves" for the venous and arterial compartments were linear such that both curves gave the mean circulatory pressure (MCP) at the unstressed volumes (V_v_0 and V_a_0, see Fig. 6). 3) That the venous pressure could not be less than zero, and that the arterial pressure could not be less than the MCP. 4) That heart rate was constrained to be between 0 and 220. 5) That the venous pressure – stroke volume relationship was non-linear and reached a plateau consistent with the finite pumping capacity of the heart (Fig. 6). 6) For convenience, two additional parameters were introduced (S1 and S2) representing the state of the sympathetic nervous system. These gave the capacity to adjust the proportionality term between blood pressure and heart rate (HRgain) and between CVP and stroke volume (kc). This allowed these scaling constants to be separated into a constant term that is solely used to convert measurement units (HRgain or kc) and another term (S1 or S2) that represents changes in underlying physiology for use when fitting data. Their normal values were 1 in each case (giving no effect for baseline conditions) and their function is summarised in the following equations:

HR = MAPset + MAPdelta*(HRgain*S1)    ...(7)



**Figure 7:**
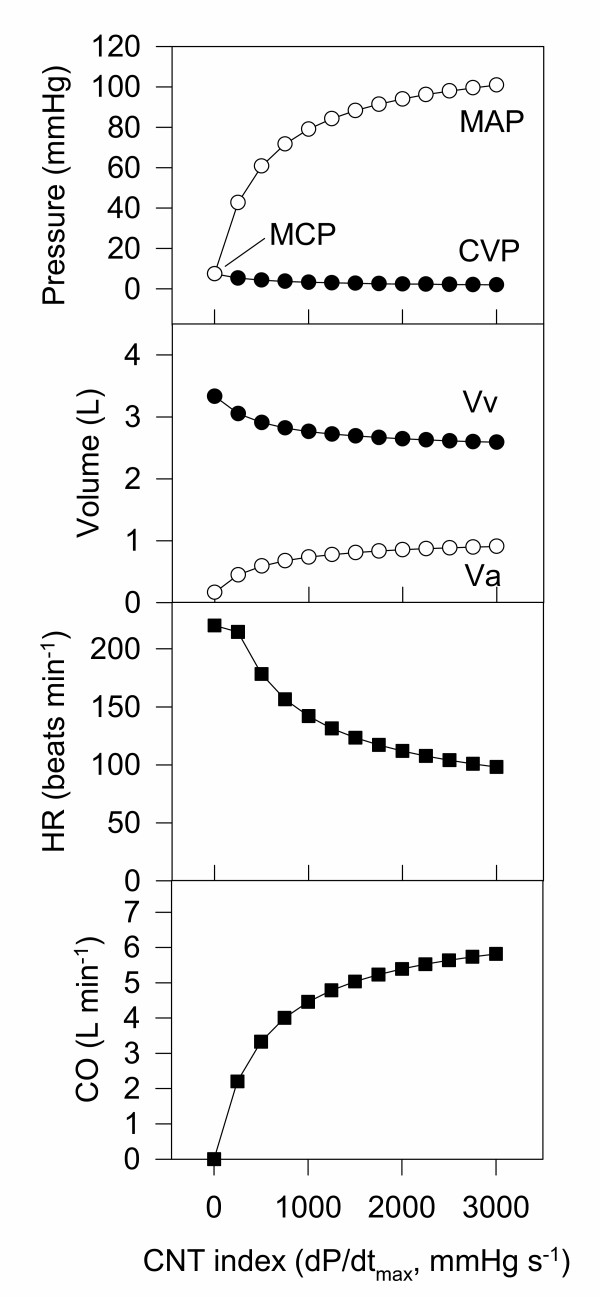
**Cardiac function curves for the Constained-Frank-Starling-Baroreceptor model. **A summary of the behaviour of the final cardiovascular dynamic model.

The resultant cardiac function curves for this model are shown in Fig. 7, and the equations for the model are shown in Additional File 3.

### Baseline values for the cardiovascular model

For convenience, the target cardiovascular variables of the final constrained model discussed above are summarised in Table 1 with references to their origins. The parameter set that produced cardiovascular variables similar to the target values is summarised in Table 2. This was derived semi-empirically by inspection of cardiovascular function curves (Fig. 7) and pressure-volume relationships (Fig. 6) with incremental adjustment of parameter values. Note that some variables are also listed as parameters – this is purely for convenience. The distinction between variables (time-dependent) and parameters (time-independent) is semantic and depends on the proposed use of the model.

**Table 2:** Baseline model parameters The parameters chosen as those producing representative baseline (pre-drug) cardiovascular variables (Table 1). The co-efficient of variation (CV (%)) of these parameter values as determined by the Monte-Carlo sensitivity analysis is also shown.

**Parameter**	**Name**	**Value**	**Units**	**CV (%)**
CPL_v_	Venous compliance	0.45	L mmHg^-1^	12.7
CPL_ratio_	Ratio of venous over arterial compliance	20	dimensionless	17.7
V_blood_	Blood volume	3.5	L	17.9
SVR	Systemic vascular resistance	17	RU	5.5
MAPset	Mean arterial pressure set point	100	mmHg	4.2
HRgain	Gain for heart rate control	1.8	bpm^a ^mmHg^-1^	23.6
CNT	Contractility	3000	mmHg sec^-1^	4.9

^a^bpm = beats per minute

The sensitivity of the baseline cardio-vascular model to changes in parameter values was determined via Monte-Carlo simulation [14]. Multi-variate normally distributed noise was added to the parameter values for a series of 10,000 simulations of the resulting cardiovascular variables. Those parameter sets that produced a set of cardiovascular variables within 10% of the target set were selected and analysed for with respect to parameter variability and correlation.

### Fitting the cardiovascular model to the magnesium data

Changes in cardiovascular variables with the administration of magnesium were analysed as percentage change from baseline. This removed the contribution of inter-animal variability in baseline cardiovascular variables (which was nevertheless minor [8,9]) to variability in the cardiovascular effects of magnesium. The analysis involved fitting cardiovascular radar plots to the measured magnesium data (Data set 1) for key time-points (1, 2, 4, 10 and 25 min) during and after magnesium administration. The cardiovascular model was parameterised in terms of primary cardiovascular variables that could be directly influenced by magnesium. These were SVR, CPL_v_, CPL_ratio_, CNT, S1 and S2. V_blood _could also be considered a primary variable, but it was considered unlikely that magnesium could change the blood volume. The remaining cardiovascular variables were considered secondary in that they would change in response to changes in the primary variables as given by Eqns 2 to 4.

Initially, the only primary parameter fitted to the data for each time point was SVR while the other parameters were held constant. This was based on the prior knowledge that this was the primary mechanism of action of magnesium. If the MSC was low and the cardiovascular radar plot showed a poor fit between model predictions and the data, an additional parameter was fitted one at a time from the remaining parameters listed above. A parameter was removed from the fit if it produced an undefined estimate. The parameter was kept in the fit if it improved the MSC and the fidelity of observed vs. predicted plots on the cardiovascular radar. By this process, the values of the primary cardiovascular parameter at each key time point required to describe the observed data were determined.

### Recirculatory pharmacokinetic model of magnesium disposition

**Figure 10:**
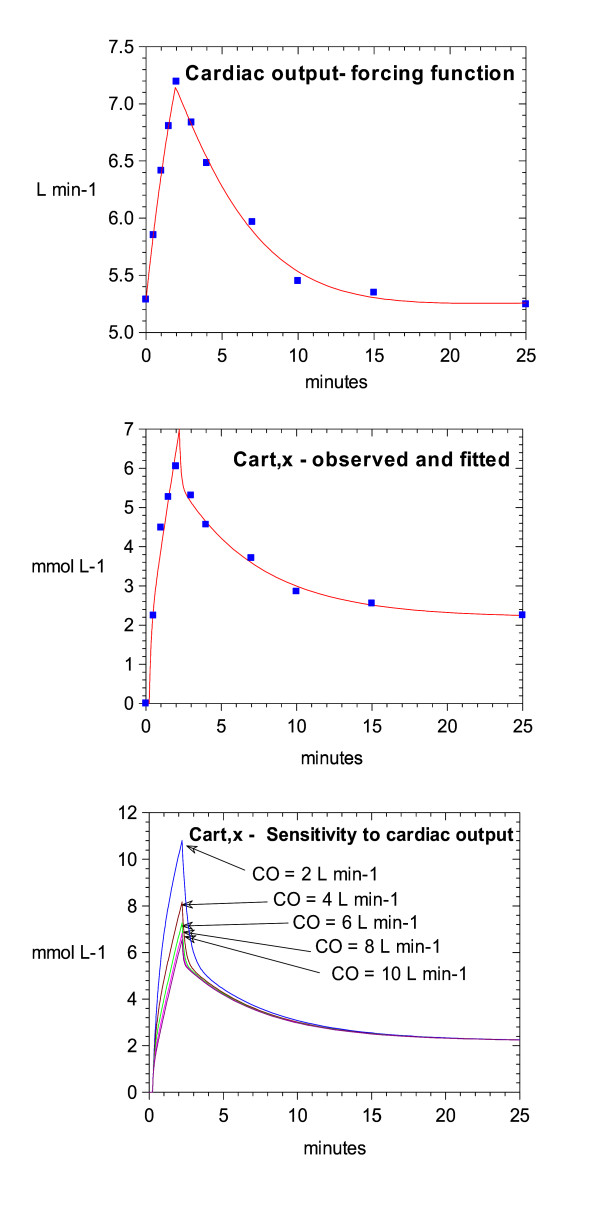
**Best fits for the recirculatory pharmacokinetic model for magnesium. **Top: The observed changes in cardiac output for Data set 1 (symbols). Also shown is the line of best fit for the empirical forcing function used for development of the kinetic model. The large and consistent increase in cardiac output illustrates why it was necessary to use a kinetic model that could account for the significant flow changes caused by magnesium. Middle: The observed arterial concentrations of magnesium for Data set 1 (symbols). Also shown is the line of best fit for the final kinetic model (not linked to the cardiovascular model) based on the parameter values given in Table 4. Bottom: A sensitivity analysis of the final kinetic model with respect to cardiac output when used to simulate the dose regimen used for Data set 1. Cardiac output was given values of 2, 4, 6, 8 or 10 L min^-1 ^while the other parameters were fixed at the values given in Table 4. This illustrates how the cardiac output changes caused by magnesium can influence its own kinetics. This feedback process was inherent in the structure of the final kinetic-dynamic model.

Conventional mamillary pharmacokinetic models are essentially empirical and do not include parameters (other than clearance) that represent defined physiological processes. This is problematic when drugs affect the cardiovascular system, or it is necessary to predict the kinetics of the drug when the underlying physiology has changed. This was the case for magnesium, which affected cardiac output significantly (Figs. 1 &10). Full physiological pharmacokinetic (PBPK) models are an alternative, but often require extensive data sets for their parameterisation. Recirculatory models have been used [7,15] as an alternative that retain the key physiological descriptions of important organs, but have lumped descriptions of the less important organs. Often, they can be parameterised by fitting blood concentrations alone.

**Figure 9:**
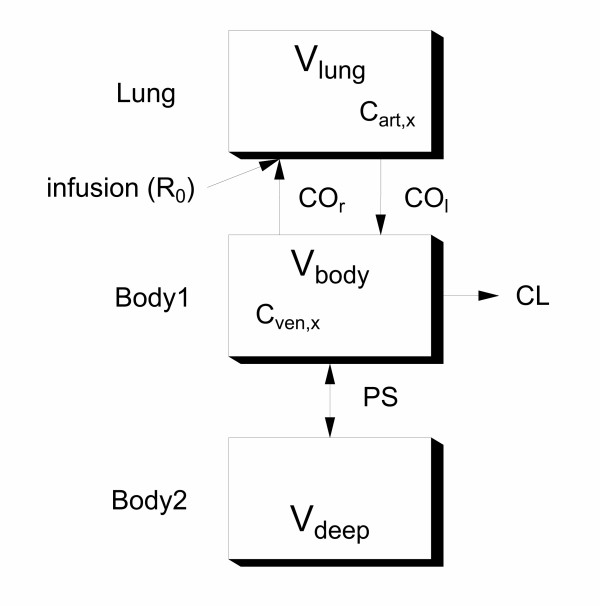
**Final recirculatory pharmacokinetic model for magnesium. **A pictorial representation of the model. Parameter names are given in Table 4.

The magnesium concentration data from Data set 1 were used to develop a recirculatory model of magnesium kinetics that could account for the observed cardiac output changes. The processed used was similar to that described by the authors for other drugs [15]. The final form of the model is shown in Fig. 9.

Key points during the model development process were: 1) The representation of the lungs as a single compartment. 2) The representation of the cardiac output change as an empirical forcing function (see Fig. 10, this would later be replaced by the predictions of the final cardiovascular model). 3) The representation of the body as extracellular and intracellular spaces connected by a permeability term, in keeping with the known slow cellular uptake of magnesium. 4) The clearance of magnesium is renal, but it can be reabsorbed or excreted in the tubules, as dictated by homeostatic requirements [16]. Thus, renal clearance may be variable.

To confirm that the kinetics of magnesium were cardiac output dependent, the final kinetic model was subjected to a sensitivity analysis for this parameter. Cardiac output was assigned values of 2, 4, 6, 8 or 10 L min^-1 ^while the other parameters were fixed at their best fit value. The time-course of the arterial magnesium concentration was recorded in each case.

### Linking the pharmacokinetic and pharmacodynamic models

The relationship between the key cardiovascular parameters (effects) and the concentrations of magnesium in arterial and coronary sinus blood were examined using hysteresis plots (effect vs. concentration). A concentration-effect relationship was considered plausible if produced a predictable relationship with minimal hysteresis that was consistent with the known mechanisms of action of the drug.

**Figure 11:**
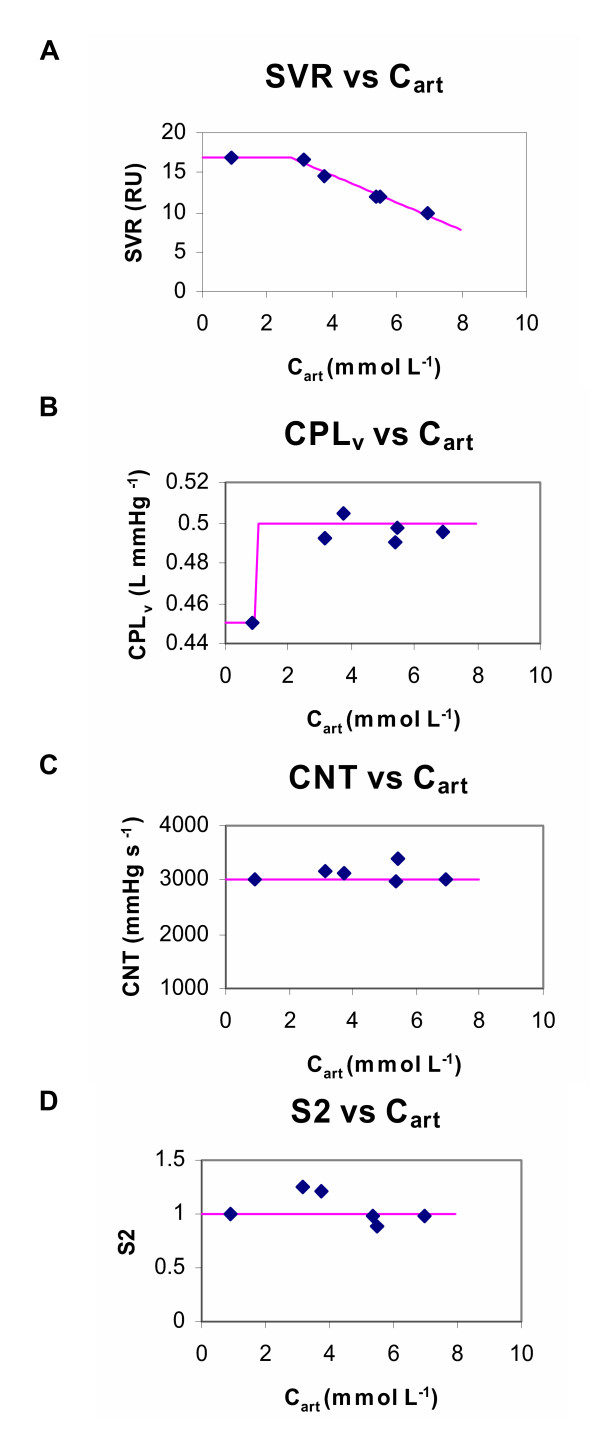
**Link models for concentration-effect relationships. **A: The systemic vascular resistance (SVR) parameter (symbols, obtained by the fitting process that gave the radar plots shown in Fig. 8 and summarised in Table 3) plotted against the concurrent exogenous arterial magnesium concentrations. The final link model (Eqn. 9; line) based on a linear relationship with a threshold is also shown. B: The venous compliance (CPL_v_) parameter (symbols, via Fig. 8) plotted against the concurrent exogenous arterial magnesium concentrations. The final link model (Eqn 10; line) based on a simple threshold that switches between two states of venous compliance is also shown. This is plausible if it is considered that magnesium, even at relatively low concentrations, causes maximal dilation of the venous capacitance vessels. C: The contractility (CNT) parameter (symbols, via Fig. 8) plotted against the concurrent exogenous arterial magnesium concentrations. The final link model (line) was based on the assumption that contractility was unaffected by magnesium (i.e parameter value was fixed). D: The sympathetic tone coefficient for contractility (S2) parameter (symbols, via Fig. 8) plotted against the concurrent exogenous arterial magnesium concentrations. The final link model (line) was based on the assumption that S2 was unaffected by magnesium (i.e parameter value was fixed).

By these criteria, it was found that the arterial concentrations were the better predictor of the fitted cardiovascular parameters shown in Table 3. The concentration – effect relationships are summarised in Fig. 11. The major effect of magnesium was to drop systemic vascular resistance (SVR). SVR was related to the arterial magnesium concentration by a link model based on a linear relationship with a threshold (Fig. 11A):

**Table 3:** The fitted primary cardiovascular parameters for Magnesium data set 1 Units are as for Table 2. The parameter estimates are given with the standard deviation returned by the curve-fitting program. S1 could not be reliably fitted to the data.

	**0 min**	**1 min**	**2 min**	**4 min**	**10 min**	**25 min**
Fitted parameter	(baseline)	estimate (sd)	estimate (sd)	estimate (sd)	estimate (sd)	estimate (sd)

MSC	n/a	3.88	2.96	4.75	1.67	4.74
CPL_v_	0.45	0.490 (0.0028)	0.495 (0.0075)	0.497 (0.0019)	0.505 (0.0049)	0.492 (0.0007)
SVR	17	11.99 (0.12)	9.84 (0.26)	11.84 (0.084)	14.64 (0.27)	16.74 (0.077)
CNT	3000	2988 (41)	3031 (104)	3388 (32)	3108 (80)	3180 (23)
S2	1	0.978 (0.023)	0.972 (0.055)	0.877 (0.014)	1.22 (0.06)	1.25 (0.017)

if C_art _< 2.66 then

SVR = 17

else

SVR = -1.759*C_art _+ 21.68    ...(9)

Magnesium also raised venous compliance (CPL_v_). This was related to the arterial concentration using a simple threshold (Fig. 11B):

if C_art _< 2 then

CPL_v _= 0.45

else

CPL_v _= 0.50    ...(10)

Magnesium had little effect on myocardial contractility (Fig. 11C), and the linking function assumed that CNT remained at baseline values. Magnesium appeared to increase the sympathetic tone coefficient for contractility (S2) by approximately 25% at between concentrations of 2 and 4 mmol L^-1 ^(Fig. 11D). However, this rise in S2 only occurred late in the study (Table 3). It indicates subtle changes in the relationship between the filling pressure index (LVEDP) and the contractility index (dp/dt). This may reflect measurement error in these variables, non-stationarity in the experimental preparation or subtle delayed changes in myocardial compliance caused by magnesium. However, it was found that a link function assuming S2 remained at baseline values (Fig. 11D) was an adequate account of the data and did not compromise the predictive power of the model in the validation stage.

**Figure 12:**
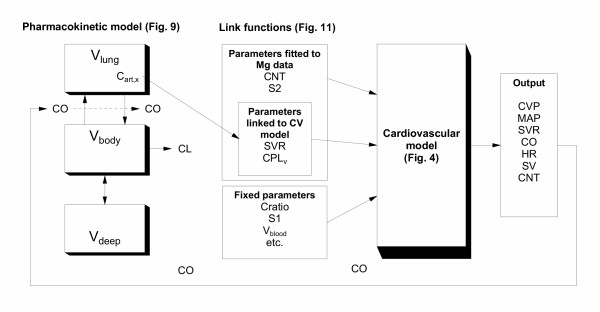
**Overview of the kinetic-dynamic model linking process. **A schematic representation of how the final model was derived from Data set 1. The pharmacokinetic (PK) component of the model was developed by fitting the observed arterial magnesium concentrations (Fig. 10, middle). As cardiac output was a parameter of the recirculatory model, the magnesium induced changes in cardiac output were represented as a forcing function during fitting (Fig. 10, top). In the final model, this forcing function was replaced by the cardiac output predicted by the cardiovascular (CV) model. For the CV model, target baseline cardiovascular variables were derived from previous measurements and the literature (Table 1). A unique parameter set for the CV model was found that reproduced these values (Table 2). To account for the changes in cardiovascular variables from baseline following magnesium, four parameters (SVR, CPL_v_, CNT and S2) were fitted to the observed magnesium CV data (expressed as change from baseline) at selected time-points (Fig. 8; Table 3). Of these, two parameters (SVR, CPLv) showed concentration dependent changes that could be related via link functions to the time-course of magnesium concentrations (Fig. 11). The other parameters of the CV model were fixed at their baseline values. The final model was able to predict the concentrations and CV effects of magnesium for a different dose regimen (Data set 2, Fig. 13).

The final kinetic-dynamic model therefore consisted of the kinetic model shown in Fig. 9 linked to the Constrained-Baroreceptor-Frank-Starling cardiovascular dynamic model (Figs. 4 &7) via the link Equations 9 and 10. This is summarised in Fig. 12. The equations for the model are shown in Additional file 4.

### Validation of the final model

The final kinetic-dynamic model developed using Data set 1 was used to predict the arterial magnesium concentrations, cardiac output and mean arterial blood pressure for Data set 2. Data set 2 differed from Data set 1 in that the dose of magnesium was given over 5 min instead of 2 min. Consequently, although the dose was the same, the cardiovascular effects were less pronounced. For example, the lowest blood mean arterial pressure for Data set 1 was 76% of baseline, while for Data set 2 this was 86% of baseline. The only change made to the parameters of the final model was to alter the duration of infusion of the magnesium.

## Results

### Parameter sensitivity of cardiovascular model (baseline conditions)

The baseline cardiovascular variables and the parameters that produced them are summarised in Tables 1 and 2, respectively. Of the 10,000 random parameter sets examined in the Monte-Carlo sensitivity analysis, only 37 produced a set of cardiovascular variables that was within 10% of the target cardiovascular variables. The variability of these successful parameter values was low (Table 2), and the spread of each parameter showed a unimodal, approximately normal distribution. This suggests that there was a unique set of parameter values for the model that was consistent with normal baseline physiology. Visual inspection showed no obvious correlation between parameter values, except for CPL_v _and CPL_ratio _(correlation coefficient = 0.83). This suggests that specifying the value for one of these parameters significantly constrains the value that can be taken for the other, as would be expected on physiological grounds. It can be concluded that each parameter had an important role to play in the model, and that each could only take a limited range of values to be consistent with the required baseline physiology. By extension, the assumptions regarding the values of these parameters are likely to be appropriate. Furthermore, the changes in these parameters observed following magnesium administration therefore reflect the effects of this drug rather than uncertainty in the parameter space of the model.

### Parameter estimates – cardiovascular data

**Figure 8:**
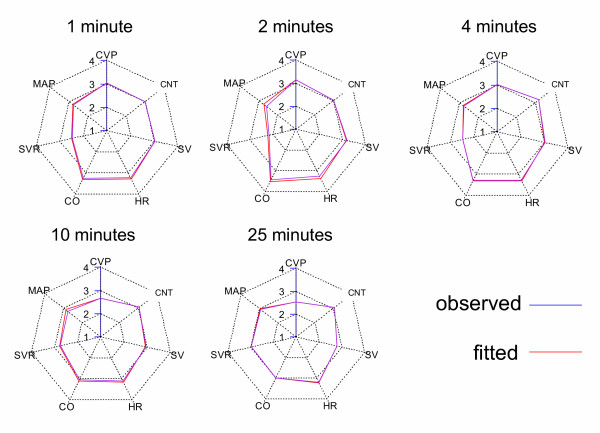
**Best fit cardiovascular radar plots for each key time point. **Cardiovascular radar plots of the observed data (blue) and the best fit of the final cardiovascular model (red). Note that the shape of the radar plot changes with time, indicating the evolving effects of magnesium on the circulation. For each time-point, the fit was an adequate account of the data (Table 3).

The method of estimating cardiovascular model parameters from cardiovascular data for individual time points was effective. Thus, it was possible to find a parameter set at each time point (Table 3) that produced a fitted cardiovascular radar plot that closely matched the observed plot (Fig. 8). In general, the parameter estimates were precise. The most obvious effect of magnesium was a drop in systemic vascular resistance and a rapid and sustained increase in venous compliance. The changes in the other cardiovascular variables (e.g. HR and MAP) simply reflected reflex changes in response to these primary drug effects.

### Parameter estimates – pharmacokinetic data

The recirculatory pharmacokinetic model was able to fit the observed concentrations with adequate fidelity (Fig. 10, middle) and produce precise parameter estimates (Table 4). As the clearance of magnesium was low, it would be expected that the permeability term into the deep compartment governed the rate of decline of the magnesium concentration rather than its clearance from the body.

**Table 4:** The fitted pharmacokinetic parameters for the Magnesium data set 1 The parameter estimates are given with the standard deviation returned by the curve-fitting program.

**Fitted variable**	**Value**	**Units**
MSC	3.13	
V_lung_	0.887 (0.221)	L
CL	0.0021 (0.1286)	L min^-1^
V_body_	4.023 (0.486)	L
PS	0.589 (0.227)	L min^-1^
V_deep_	8.63 (5.39)	L

A feature of recirculatory pharmacokinetic models is that their initial kinetics are governed by first-pass passage of drug through the lungs, and the dilution of the injected drug with the cardiac output [7]. The cardiac output sensitivity analysis for the model confirmed this behaviour for magnesium (Fig. 10, bottom). This reinforces the need for a common cardiac output term for the cardiovascular and recirculatory kinetic model (Fig. 12). The resultant final model therefore accounts for the fact that by altering cardiac output, magnesium alters its own kinetics.

### Link functions

Relating the estimated cardiovascular parameters in Table 3 to the concurrent arterial concentrations produced the concentration-effect curves shown in Fig. 11. Link functions were established for SVR and CPLv, but not CNT or S2. The overall role of the link functions is summarised in Fig. 12.

### Model validation – pharmacokinetic component

**Figure 13:**
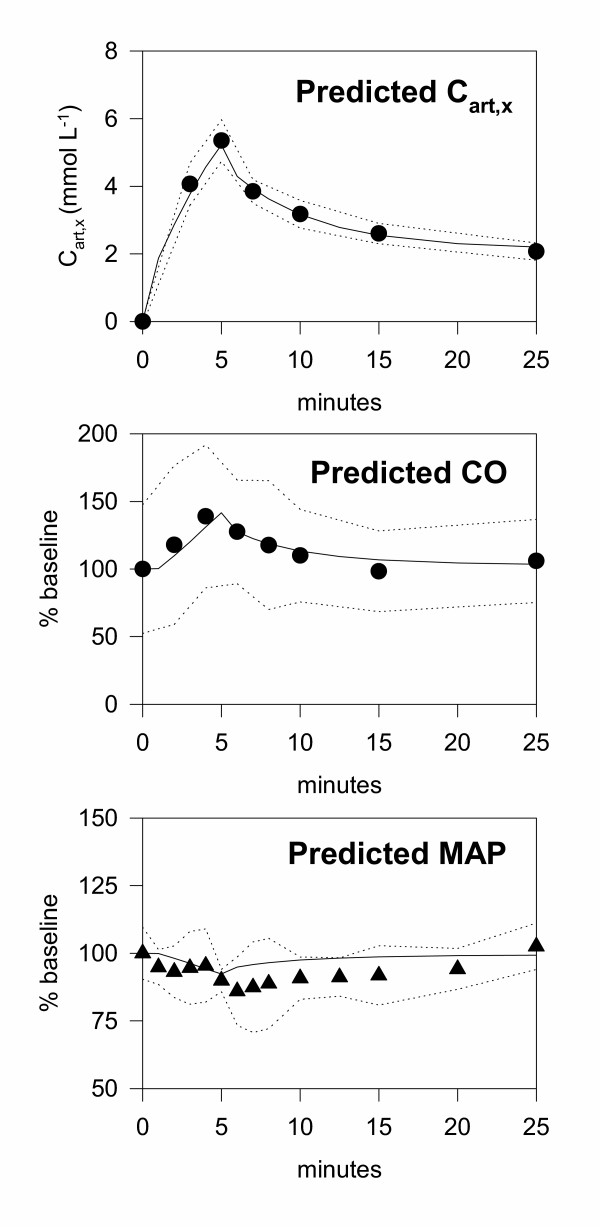
**Observed and predicted results for Data set 2. **The final kinetic-dynamic model developed using Data set 1 was used to predict the exogenous arterial magnesium concentrations (C_art,x_), cardiac output (CO) and mean arterial pressure (MAP) for Data Set 2. The only change in the model between the two data sets was to increase the duration of the infusion from 2 to 5 min. The observed data are shown as symbols, together with the upper and lower 95% confidence intervals of the data (dotted lines). The predictions of the model are shown by the solid lines.

The recirculatory model of magnesium disposition was able to accurately predict the time-course of the arterial magnesium concentrations observed for the validation Data set 2, despite the large change in cardiac output produced by magnesium (Fig. 13). The mean prediction error was 0.02%

### Model validation – pharmacodynamic component

The final pharmacodynamic model was able to accurately predict the time-course of the cardiac output changes observed for the validation Data set 2 (Fig. 13). The mean prediction error was 3.0%.

The dynamic model captured the general trend of the mean arterial blood pressure for the validation data (Fig. 13), but some systematic deviations were evident. The model was accurate until the end of the infusion, but thereafter slightly over-estimated the rate of recovery of blood pressure. However, the model did predict that the drop in blood pressure would be considerably less for a 5 min versus 2 min infusion, and the overall magnitude of the changes in blood pressure for the 5 min infusion were small (less than 10% change). The mean prediction error was 6.1%.

## Discussion

### Concentration-effect relationships and recirculatory models

In this paper, all cardiovascular effects were related to the arterial concentration of magnesium. As covered in the introduction, there may be other sites in the body that have a theoretical claim to being the most appropriate link concentration for certain cardiovascular dynamic effects. For example, the reductions in myocardial contractility caused by thiopental have been shown to have a better temporal relationship to the thiopental concentrations in the myocardium itself rather than in arterial blood [17]. This consistent with a direct thiopental effect on the myocardium.

In recirculatory models, it is possible to add a "target organ" to represent organs such as the heart [18]. The fact that this was not necessary for magnesium may be the exception rather than the rule. As magnesium has small volumes of distribution, there is little difference in the time-course of the arterial and regional venous concentrations. Furthermore, the predominant effects of magnesium were directly on blood vessels (arterioles for SVR and large veins for capacitance) in direct equilibrium with blood rather than organs such as the heart or brain. Thus, a "systemic" recirculatory model was sufficient for magnesium. As other drugs are studied using this method, data on target organ kinetics and their incorporation into the kinetic model may be necessary.

### Limitations

There are a number of limitations of this modelling approach, many of which are inherent in the assumptions made in the construction of the model. Other limitations may become apparent if the model is used outside of the range of the data used to develop the model. For example, the CL term in the kinetic model was very low (Table 4). This may reflect extensive tubular re-absorption, but may also reflect the fact that the concentrations were followed for only 25 min in the original paper (the time by which most cardiovascular variables had returned to baseline). Studies of a longer duration would help to define this clearance term better.

The cardiovascular model also assumes an instantaneous baroreceptor response. While it is relatively easy (in modelling terms) to add a delay to this response, this was not supported by the data. However, if the model is extended to situations with very rapid blood pressure changes (e.g. orthostatic hypotension) this deficiency may become significant.

Constructing physiologically based models, even of the simplicity presented here, requires crossing many decisions points where a choice must be made from multiple options – sometimes the choices are data driven, sometimes theory driven, sometimes the subjective experience of the model maker must be called upon. While a "wrong" model is evident because it does no match the data, there is clearly no "right" model of the cardiovascular system. It is anticipated that more limitations of the cardiovascular dynamic model will become apparent when model is rigorously compared to data for other drugs, and for other cardiovascular scenarios. It is should be expected that the model will continue to evolve as these data are collected and analysed.

## Conclusion

The combination of the recirculatory kinetic model and the simple cardiovascular dynamic model was able to describe and predict the concentrations and cardiovascular effects of magnesium in sheep. It is proposed that the general methods used here could be applied to other drugs with cardiovascular effects. The authors are currently applying the method to intravenous anaesthetics.

## Abbreviations

**Cardiovascular term Description**

Frank-Starling model

V_blood _Blood volume

CVP = P_v _Central venous pressure

MAP = P_a _Mean arterial pressure

MCP Mean circulatory pressure

V_a _Volume of blood in arterial compartment

V_v _Volume of blood in venous compartment

V_a_0 Volume of blood in arterial compartment at MCP

V_v_0 Volume of blood in venous compartment at MCP

CPL_a _Arterial compliance

CPL_v _Venous compliance

CPL_ratio _Ratio of venous over arterial compliance

SVR Systemic vascular resistance

CO Cardiac output

CO_L _Cardiac output (left side)

CO_R _Cardiac output (right side)

HR Heart rate

SV Stroke volume

kc unit conversion factor – contractility

CNT Contractility

additional for Frank-Starling-Baroreceptor model

MAPset Mean arterial pressure set point

HRgain Gain for heart rate control

additional for Constrained-Frank-Starling-Baroreceptor model

P_a_S Pressure in arterial compartment when stressed

P_v_S Pressure in venous compartment when stressed

V_a_S Volume in arterial compartment when stressed

V_v_S Volume in venous compartment when stressed

slopeMAP slope for arterial pressure-volume relationship

intMAP intercept for arterial pressure-volume relationship

slopeCVP slope for venous pressure-volume relationship

intCVP intercept for venous pressure-volume relationship

S1 Sympathetic tone coefficient – Chronotropy

S2 Sympathetic tone coefficient – Contractility

SV_max _maximum for stroke volume-CVP relationship

SV_50 _half-volume for stroke volume-CVP relationship

nSV "Hill factor" for stroke volume-CVP relationship

**Pharmacokinetic term Description Description**

Recirculatory model

R_0 _doserate of zero order infusion

tau duration of zero order infusion

C_art _Arterial magnesium concentration (total)

C_ven _Venous magnesium concentration (total)

C_art,e _Arterial magnesium concentration (endogenous)

C_ven,e _Venous magnesium concentration (endogenous)

C_art,x _Arterial magnesium concentration (exogenous)

C_ven,x _Venous magnesium concentration (exogenous)

V_lung _Apparent distribution volume of the lung

CL Clearance

V_body _Apparent distribution volume of the body compartment

PS Permeability-surface area product of deep compartment

V_deep _Apparent distribution volume of the deep compartment

## Authors' contributions

RNU participated in the original magnesium studies and performed the modelling. GLL participated in the original magnesium studies and acted as a resource for cardiovascular theory. Both authors contributed to the manuscript.

## Supplementary Material

Additional File 1 The simple Frank-Starling model. The simple Frank-Starling model written in pseudo-code to generate cardiac function curves. The code is intended to run in the "Scientist" differential equation solving program. Click here for file

Additional File 2 The Frank-Starling-Baroreceptor model. The equations for the Frank-Starling-Baroreceptor model with baroreceptor control written in pseudo-code to generate cardiac function curves. Click here for file

Additional File 3 The Constrained Frank-Starling-Baroreceptor model. The equations used for the final cardiovascular model written in pseudo-code to generate cardiac function curves. Click here for file

Additional File 4 The final pharmacokinetic-pharmacodynamic model. The equations used for the final model written in pseudo-code. Click here for file

## References

[B1] Coleman TG (1985). Mathematical analysis of cardiovascular function. IEEE Transactions on Biomedical Engineering.

[B2] Kappel F, Peer RO (1993). A mathematical model for fundamental regulation processes in the cardiovascular system. J Math Biol.

[B3] Smith NT, Zwart A, Beneken JEW (1972). Interaction between the circulatory effects and the uptake and distribution of halothane: Use of a multiple model. Anesthesiology.

[B4] Fukui Y, Smith NT (1981). Interactions among ventilation, the circulation, and the uptake and distribution of halothane – use of a hybrid computer multiple model: I. The basic model. Anesthesiology.

[B5] Tham RQY, Sasse FJ, Moller DPF (1990). Large-scale multiple model for the simulation of anesthesia. Advanced simulation in biomedicine.

[B6] Francheteau P, Steimer JL, Merdjan H, Guerret M, Dubray C (1993). A mathematical model for dynamics of cardiovascular drug action: application to intravenous dihydropyridines in healthy volunteers. J Pharmacokinet Biopharm.

[B7] Upton RN (2004). The two-compartment recirculatory model – an introduction to recirculatory pharmacokinetic concepts. Br J Anaesth.

[B8] Ludbrook GL, James MF, Upton RN (1999). The effect of magnesium sulfate on cerebral blood flow velocity, cardiovascular variables, and arterial carbon dioxide tension in awake sheep. J Neurosurg Anesthesiol.

[B9] Zheng D, Upton RN, Ludbrook GL, Martinez A (2001). Acute cardiovascular effects of magnesium and their relationship to systemic and myocardial magnesium concentrations after short infusion in awake sheep. J Pharmacol Exp Ther.

[B10] Huang YF, Upton RN, Runciman WB (1993). IV bolus administration of subconvulsive doses of lignocaine to conscious sheep: relationships between myocardial pharmacokinetics and pharmacodynamics. Br J Anaesth.

[B11] Ludbrook GL, Upton RN, Grant C, Gray EC (1996). Brain and blood concentrations of propofol after rapid intravenous injection in sheep, and their relationships to cerebral effects. Anaesth Intens Care.

[B12] Ihaka R, Gentleman R (1996). R: A language for data analysis and graphics. J Comput Graphical Stat.

[B13] Torrington KG, McNeil JS, Phillips YY, Ripple GR (1989). Blood volume determinations in sheep before and after splenectomy. Lab Anim Sci.

[B14] Bernillon P, Bois FY (2000). Statistical issues in toxicokinetic modeling: A bayesian perspective. Environ Health Perspect.

[B15] Upton RN, Ludbrook GL (1997). A physiological model of induction of anaesthesia with propofol in sheep. 1. Structure and estimation of variables. Br J Anaesth.

[B16] Yucha C, Dungan J (2004). Renal handling of phosphorus and magnesium. Nephrol Nurs J.

[B17] Huang YF, Upton RN, Gray EC, Grant C, Zheng D, Ludbrook GL (1997). The effects of short intravenous infusions of thiopentone on myocardial function, blood flow and oxygen consumption in sheep. Anaesth Intensive Care.

[B18] Huang YF, Upton RN, Zheng D, McLean C, Gray EC, Grant C (1998). The enantiomer-specific kinetics and dynamics of verapamil after rapid intravenous administration to sheep: physiological analysis and modeling. J Pharmacol Exp Ther.

[B19] Guyton AC, Hall JE, Arthur C Guyton, John E Hall (1982). Cardiovascular physiology IV.

[B20] Stalberg HP, Hahn RG, Hjelmqvist H, Ullman J, Rundgren M (1993). Haemodynamics and fluid balance after intravenous infusion of 1.5% glycine in sheep. Acta Anaesthesiol Scand.

[B21] Huang YF, Upton RN, Rutten AJ (1996). Adverse haemodynamic effects of the rapid intravenous injection of hypotonic solutions in sheep. Res Vet Sci.

